# Dissociative Disorders: Between Neurosis and Psychosis

**DOI:** 10.1155/2014/425892

**Published:** 2014-10-22

**Authors:** C. Devillé, C. Moeglin, O. Sentissi

**Affiliations:** Department of Mental Health and Psychiatry, General Psychiatry Department, University Hospital of Geneva, CAPPI Jonction, Rue des Bains 35, 1205 Geneva, Switzerland

## Abstract

Dissociative disorders are a set of disorders defined by a disturbance affecting functions that are normally integrated with a prevalence of 2.4 percent in industrialised countries. These disorders are often poorly diagnosed or misdiagnosed because of sharing common clinical features with psychotic disorders, but requiring a very different trajectory of care. Repeated clinical situations in a crisis centre in Geneva provided us with a critical overview of current evidence of knowledge in clinical and etiopathological field about dissociative disorders. Because of their multiple expressions and the overlap with psychotic disorders, we focused on the clinical aspects using three different situations to better understand their specificity and to extend our thinking to the relevance of terms “neurosis” and “psychosis.” Finally, we hope that this work might help physicians and psychiatrists to become more aware of this complex set of disorders while making a diagnosis.

## 1. Introduction

Dissociative disorders are a complex syndrome because of multiple expressions and the wide variety, defined by disturbances of every area of psychological functioning, affecting functions that are normally integrated such as memory, consciousness, identity, emotion, perception, body representation, motor control, and behaviour [1].

Major changes in dissociative disorders in the recent fifth edition of DSM-5 include the following: (1) derealization is included in the name and symptom structure of what previously was called depersonalization disorder (depersonalization-derealization disorder); (2) dissociative fugue is now a specifier of dissociative amnesia rather than a separate diagnosis; and (3) the criteria for dissociative identity disorder were changed to indicate that symptoms of disruption of identity may be reported as well as observed and that gaps in the recall of events may occur for everyday and not just traumatic events. Also, experiences of pathological possession in some cultures are included in the description of identity disruption.

According to ICD-10, there are more subtypes of diagnostic categories and depersonalization/derealization disorder is classified in neurotic disorders (see Table 1).

These classifications admit that dissociative disorders are psychogenic, that is, of purely mental origin [2]. At the present time, experts on this field agree that classifications and definitions of this disorder are insufficient [3].

The prevalence of dissociative disorders is close to 2.4 percent in industrialised countries [4] and, for dissociative identity disorder, the prevalence is close to 1 percent [1]. The authors believe that these results are often undervalued [5]. The sex ratio is 1 : 1 [6].

Diagnostic of dissociative disorders can overlap with psychotic disorders, reflecting the close relationship between these diagnostic classes [7–11]. This may contribute to diagnostic errors and therefore lead to inadequate care and treatment management.

The history of the concept of dissociation goes back to the works of Charcot and Bernheim on hysteria and hypnosis and then those of Janet and Freud. With Bleuler, the concept of “dissociation” extends and is soon permanently reduced to some symptoms of schizophrenia, known from clinicians as “Spaltung,” a psychic disintegration expressed in discordant manifestations of thoughts, affects, and behaviour. This division contributes, even at the present time, to supply issues on the border, sometimes blurred, between hysterical symptoms, posttraumatic stress, and schizophrenia.

“The dissociation would focus on the body representation, in the direction of a separation of body and psyche (…)” [12]. Dissociative disorders correspond to a less archaic way than schizophrenia, with an important sensory oppression component recognised by the evoking apprehended foreign sensations [12]. Laferrière-Simard and Lecomte [10] mention authors, including Janet (1894), Follin, Chazaud and Pilon (1961) who suggest the terms of madness and hysterical psychosis. Freud sometimes describes psychosis as an aggravated neurosis and Henry Ey thinks of neurosis as “a first degree of fall in psychosis” [12]. In 1993, van der Hart et al. [13] suggest the term of dissociative reactive psychosis, instead of hysterical psychosis, diagnosed when an immersion in phenomena of traumatic origin becomes invasive for the patients. The psychotic characteristics would decrease or disappear when the traumatic origins are identified. In 2004, Ross and Keyes [14] suggest the existence of a distinct group of people who suffer from schizophrenia, with dissociation as the underlying expression of psychotic symptoms and, in this sense, they propose to create the subtype of dissociative schizophrenia like the paranoid or the catatonic subtypes [10]. We have therefore found, through the history of hysteria, that the terms psychosis and hysteria are contained in a single concept, to mention hysterical psychosis (in ICD-10, dissociative disorder conversion is also called “hysterical psychosis”). Since the 2000s, the new concept of dissociative schizophrenia emerges. So we have noticed that the term dissociative is once associated with neurosis and once with psychosis, or even both.

Moreover the dissociative disorders are frequently found in the aftermath of trauma, correlated or not with the emotional life during childhood [15, 16]. This latter consideration, shared by dissociative disorders and schizophrenia [17], reinforces the communal phenomenological aspects and complicates the differentiation between these two clinical entities. Many of the symptoms, including embarrassment and confusion about the symptoms or desire to hide them, are influenced by the proximity to trauma. In DSM-5, the dissociative disorders are placed next to, but are not part of, the trauma- and stressor-related disorders, also reflecting the close relationship between these diagnostic categories. Both acute stress disorder and posttraumatic stress disorder contain dissociative symptoms, such as amnesia, flashbacks, numbing, and depersonalization/derealization.

We have evaluated and managed several clinical cases of dissociative disorders in the crisis centre of area-catchment of Jonction in Geneva, each one with distinct causes. To refine the diagnosis and optimise the care management of these clinical cases, we have performed a critical overview of current computerized evidence of knowledge (Medline).

## 2. Clinical Vignettes

### 2.1. Clinical Vignette Number 1

Mr. A is a 32-year-old patient of Swiss origin. He works as an insurer. He has a partner whom he has been with for over 2 years and with whom he had a child. He talks about sexual abuse from one member of his own family members in the past but has only vague memories of this event. A diagnosis of paranoid schizophrenia was established 6 years ago, and the patient has been in remission for 5 years without antipsychotic treatment. The patient has contacted us to request a diagnostic evaluation in the context of a development.

With regard to mental status, the patient is calm and collaborating; his thoughts have an organised structure; he is well-oriented, and his hygiene and clothing are appropriate. His thymia is neutral and there are no elements of depressive symptomatology. His speech is coherent, fluid, and informative without delusional elements. His only “psychosis-like” symptomatology is the “voice hearings” in the form of voices that speak to him from within. He determines that these voices are coming from his own imagination.

Indeed, he describes constant oscillations between the presence of two distinct personalities, which he manages to differentiate. The first personality is described as that of a junkie (if he does not control himself, he lives as a person who needs to consume drugs and he goes into hiding in uninhabited buildings), and the other personality is described as that of a conformist modern man (i.e., clean looking, “well thinking,” and conforming to society's standards; an attitude he adopts elsewhere, at work, for example).

His mental status reveals the characteristics of a dissociative identity disorder. There are two distinct identities or “States of personality” in this patient; they take turns at controlling the behaviour of the patient. The disturbance is not due to the direct effects of a substance or a general medical condition. Moreover, he does not have psychotic symptomatology. He describes that the voices are coming from the inside of himself (each of the personalities interacts with him, alternately). He has no other comorbid disorder. He has one meeting a month for supportive psychotherapy. He is not treated with psychotropic medication.

### 2.2. Clinical Vignette Number 2

Mrs. B is a 44-year-old patient who has been married for 24 years; she lives with her husband and their 2 teenage children. She has no known psychiatric history. The authority of parenting has been a traumatic experience, and she has a self-assertion deficit.

She consulted the psychiatric emergency department in 2012, accompanied by her family. She presented with a behaviour disorder of gradual emergence, in the form of psychomotor agitation and “sexual” exhibition. She also had voice hearings (she hears from “an angel” coming from inside that predicts upcoming events and guides her). The self-criticism is retained. The emergency psychiatrist felt that this was a psychotic disorder not otherwise specified; he administered an anxiolytic medication (lorazepam) to quickly tranquilise the patient and transferred her to the crisis centre. Upon admission, the patient had significantly intense anxiety, had a situational mild to moderate spatiotemporal disturbance, and was confused. Her mood was sad, with minor anhedonia and minor abulia. She had a sleep disorder for three days, with insomnia at the beginning and at the end of the night. Her speech was coherent, informative, fluid, and critical in the aftermath (she says that she hears the voice of an angel, which she identifies as a production of her own imagination). Considering the persistent “psychosis-like” and mass anxiety symptomatology, antipsychotic treatment with olanzapine was administered, and it was recommended that the patient stays a few nights in the centre for further care. The presence of a comorbid depressive disorder (MADRS scale score of 19) led us to prescribe an antidepressant treatment, trazodone; the dose was increased gradually to 200 mg per day. The “psychosis-like” symptomatology started improving quickly, within 48 h, and the antipsychotic treatment was stopped. The patient was able to return home after 3 days and was followed up every week with two interview sessions. During her followup, thymic improvement was noted, with a return of the vital impetus and a decrease in the anxiety but with the emergence of a diffuse painful syndrome. Her treatment is one-session psychotherapy per week and trazodone 200 mg per day.

### 2.3. Clinical Vignette Number 3

Mrs. C is a 33-year-old patient who is a law graduate. She is married and does not have any children. She presented with a major depressive disorder of moderate intensity, generalised anxiety, and a history of alcohol dependence (having been sober for a few months). She was hospitalised for the first time in the psychiatric department for 10 days, a few weeks before we met her, due to a diagnosis of “acute and transitory psychotic disorder” (with voice hearings and a behavioural disorder that has medicolegal impacts), which has been linked to disulfiram treatment; the evolution of this disorder has been favourable with olanzapine 10 mg/day and then quetiapine 200 mg/day, in addition to the usual treatment of venlafaxine 75 mg/day. Subsequently, this patient was treated in our ambulatory unit, where risperidone 1 mg/day was prescribed, and then she was hospitalised again in the psychiatric clinic for one month. Venlafaxine was replaced by escitalopram. The dose of escitalopram was decreased to 30 mg/day as a result of an increase in her liver enzymes. We also substituted pregabalin for olanzapine 5 mg/day (which was reintroduced during the 2nd hospitalisation), because of increased feelings of depersonalization-derealization, which means a feeling of “getting out of her body,” which she described “as if” she was an automaton and having recurring feelings of being detached from herself. The patient had an improvement in her depressive symptomatology (MADRS score of 32 at admission and 12 over the course of treatment) under escitalopram 30 mg/day and pregabalin 200 mg/day. However, there was a persistence of moderate anxiety. She did not have any psychotic symptomatology. She benefitted from analytical psychotherapy with one meeting per week.

## 3. Discussion

The growing clinical interest in the different forms of dissociative disorders has led us to carry out a brief review of the literature, supported by three clinical cases to highlight this complex disorder. Dissociative disorders are difficult to distinguish from psychotic disorders not only because of the close proximity of phenomenological elements but also because of a linked aetiology due to trauma, triggering sometimes both disorders. This is further complicated by other comorbid disorders, which are often present. Authors have reported association with an anxiety disorder [18–21], a depressive state [19, 20], a borderline personality disorder, PTSD, or substance abuse (in 83 to 96% of cases of dissociative identity disorders) [1] and comorbid somatoform disorders (headache, in 79 to 91% of cases of dissociative identity disorders, conversion syndromes, and somatoform disorders in 35 to 61% of cases of dissociative identity disorders) [1].

We noted that Mrs. B presented conversion symptoms (formerly classified as hysterical), which were theatrical (there was powerful staging in front of her family) with sexual thematic (showing off nude in front of her close relations and people in her immediate environment), and she had voice hearings (pseudohallucinations) [22, 23]. The latter were described as arising from the inside (and not from the outside); in fact the morbid conscience was retained; she criticised these voices by explaining they were produced by her imagination. This patient reported becoming an outside observer of her own body with a sense of being in a dream while maintaining an intact appreciation of reality, after following treatment and with a refinement of diagnostic criteria. These symptoms and their clinical and therapeutic progression (she had good anxiolysis with lorazepam) helped us to diagnose a specified dissociative disorder. The developed diagnostic could have led us to make an incorrect diagnosis of a brief psychotic disorder if we had not investigated for the presence of dissociative disorder. The diagnosis of dissociative disorder had a real impact on the patient's treatment.

However, there are only limited data on the effectiveness of drug treatments for dissociative disorders. The psychopharmacological approach is the foremost treatment based on the presence of other comorbidities. Selective serotonin reuptake inhibitors (SSRIs) treatment allows for the reduction of comorbidities, such as anxiety and depressive symptoms, although SSRIs have little effect on the dissociative disorder itself. We treated the patient with an antidepressant to reduce both the depressive and anxiety symptomatology and the pains associated with the symptoms. Psychotherapeutic support was given in the form of psychodynamic and systemic inspiration.

The symptoms Mr. A presented were likely to generate a diagnostic error, being the differential diagnosis between a psychotic disorder and a dissociative disorder close in this case. We established a diagnosis of dissociative identity disorder for this patient, who was previously diagnosed with schizophrenia. In fact, 25 to 50% of people diagnosed with a dissociative disorder are already affected by schizophrenia [24]. Voice hearings, for example, are found in 73% of schizophrenia cases [25] and in 82 to 87% of dissociative identity disorder cases [26]. In a 2005 paper, Kluft [27] describes that, for people suffering from a dissociative identity disorder, 80% of cases perceive their voice hearings as coming from inside of themselves (pseudohallucinations), whereas, for people suffering from psychosis, 80% of cases perceive their voice hearings as coming from an external source (auditory hallucinations). Mr. A's medical files stated that he never had a disruption of behaviour nor significant delirium for a period longer than one month. This is important because these patients tend to spend more time in the health care system. In fact, they have a diagnosis and treatments which are often poorly adapted [28]. This patient did not accept a psychoactive treatment. In this case, a supportive effective therapy with attentive listening was the adequate treatment without comorbidity.

Concerning the treatment of Mrs. C, she had received a diagnosis of acute and transitional psychotic disorder treated with an antipsychotic treatment. However, this was called into question due to the traced history of the postcrisis symptomatology. She described feeling detached from herself, of “getting out of her own body,” she described voices heard internally (pseudohallucinations), and she retained morbid conscience, in the context of mass anxiety. These elements enabled us to diagnose a depersonalization-derealization disorder, which is a dissociative disorder according to DSM-5 but which is considered as a neurotic trouble in ICD-10. Concerning patients with depersonalization-derealization, they frequently use the expression “it is as if” [29, 30] to describe the state of their symptomatology. She presented comorbid disorders: a major depressive disorder associated with a generalised anxiety.

This patient received treatment with pregabalin for generalised anxiety and a selective serotonin reuptake inhibitor (escitalopram) for major depressive disorder but received no other treatment for the depersonalization-derealization disorder. Antipsychotic drugs are sometimes used to treat the depersonalization-derealization disorder; however, their effectiveness has not been demonstrated in any controlled study, and the emergence of depersonalization-derealization has been reported under antipsychotics [31, 32]. It is possible that the antipsychotic treatment she received previously could have enhanced this syndrome subsequently. The psychotherapy established for this patient was based on both the psychodynamic and systemic approaches.

The therapeutic approaches used for dissociative disorders correspond to the three basic models: cognitive-behavioural, psychodynamic, and systemic therapy. Psychotherapeutic treatments, which appear to be the most effective so far, are the EMDR [33], the psychodynamic approach [33, 34], and attentive listening to the words of the patient [34]. A few systemic approaches (of narrative inspiration, e.g.) provide interesting perspectives [35].

We assume that it is important to distinguish voice hearings experiences coming from inside (pseudohallucinations) in the dissociative disorder from those coming from outside (auditory hallucinations) in psychosis.

We have identified that dissociative disorders are a kind of trouble close to psychotic disorders because of voice hearings experiences inter alia. The “psychosis-like” symptoms (behavioural disorders, agitation, (auditory) pseudohallucinations, and pseudodelusions) are a part of dissociative disorder, giving this diagnosis hard to make. Other “psychosis-like” symptoms are the confusion and the impression to be in a “dream,” to be detached from feelings and to live something “as if.” We are aware that this is specific of depersonalization-derealization disorder, a dissociative disorder according to the DSM-5.

Finally, the specific symptoms we described in this paper allowed us suggesting that dissociative disorders are a set of troubles at the border between neurosis and psychosis. The main question of this work was to know if the dissociative disorders belong to the group of neurosis or to the one of psychosis. Are they on the border between these two entities as the clinical symptomatology and the history show us? The fact that this disorder frequently appears among patients, especially with a borderline personality disorder, points the argumentation of this discussed border leading to prospects for theoretical model of dissociative personality structure [36]. If we agree that dissociative disorder shares the same concept of hysteria which is a neurosis, that ICD-10 mentions the term “hysterical psychosis,” and also that depersonalization-derealization disorder is considered as a neurotic disorder even when we identify that it presents “psychosis-like” symptoms, this means that there is neurosis in psychosis and vice versa and thus that dissociative disorders are a separate entity. Concerning perspectives theories, it would be interesting to develop the idea that neurosis and psychosis are precarious terms, as the boundary between both is becoming increasingly blurred.

## 4. Conclusion

Adequate and well-adapted therapeutic treatment for these clinical cases of dissociative disorders has resulted in a favourable outcome in our crisis centre. We have identified that dissociative disorders are a kind of trouble close to psychotic disorders on one hand, because of voice hearing experiences inter alia, and close to neurotic disorders on the other hand, because of intact reality testing inter alia. We therefore suggest keeping focus on descriptive clinical symptomatology in this case. Further clinical studies, theoretical approaches, and reflections about this complex disorder are suitable.

## Figures and Tables

**Table 1:** Classification of dissociative disorders in ICD-10 and DSM-5.

ICD-10		DSM-5	
F44.0	Dissociative amnesia	300.12	Dissociative amnesia without fugue
F44.1	Dissociative fugue	300.13	Dissociative amnesia with dissociative fugue
F44.2	Dissociative stupor		
F44.3	States of obsession and dissociative trance		
F44.4	Dissociative motor disorders		
F44.5	Attacks of cramps dissociative		
F44.6	Sensitivity disorders and dissociative sensory		
F44.7	Mixed dissociative disorders		
F44.8	Other dissociative disorders		
F44.80	Ganser's syndrome		
F44.81	Multiple personality	300.14	Dissociative identity disorder
F44.89	Other specified dissociative disorders	300.15	Other specified dissociative disorders
F44.9	Unspecified dissociative disorders	300.15	Unspecified dissociative disorders
F48.1	Depersonalization/derealization disorder (up to neurotic disorders)	300.6	Depersonalization disorder (up to dissociation disorders)
